# Bupropion Showed Neuroprotective Impacts Against Cerebral Ischemia/Reperfusion Injury by Reducing Oxidative Stress and Inflammation

**DOI:** 10.5812/ijpr-156838

**Published:** 2025-01-04

**Authors:** Saeid Baba Ahmadi, Zeinab Afrand Khalilabad, Seyedeh Sepideh Alemohammad, Hasan Yousefi Manesh, Alireza Abdollahi, Farahnaz Jazayeri, Seyyedeh Elaheh Mousavi

**Affiliations:** 1 Department of Pharmacology, School of Medicine, Tehran University of Medical Sciences, Tehran, Iran; 2 Department of Pathology, School of Medicine, Imam Khomeini Hospital Complex, Tehran University of Medical Sciences, Tehran, Iran

**Keywords:** Bupropion, Ischemia, Rat, Cytokine, Interleukin

## Abstract

**Background:**

Cerebral ischemia/reperfusion (I/R) injury is the most prevalent form of brain stroke, affecting many patients worldwide. It is believed that oxidative stress and inflammation play major roles in the damage that occurs after the initiation of the disease.

**Objectives:**

Therefore, for the first time, the current study aimed to investigate the neuroprotective effects of bupropion against cerebral I/R damage in a rat model.

**Methods:**

Forty male rats were divided into four groups: Control, cerebral I/R, and two diseased groups that received 60 and 100 mg/kg of bupropion. One day after induction of the disease, behavioral tests, including grid walking, novel object recognition, and modified neurological severity score (mNSS), were performed on the rats. The levels of inflammatory cytokines, including IL-1β, TNF-α, IL-6, and IL-10, were measured in the rats' brain homogenates. Additionally, the levels of MDA, catalase (CAT), superoxide dismutase (SOD), reduced glutathione (GSH), and NO_2_^-^ were measured.

**Results:**

Bupropion administration was associated with improved performance in the novel object recognition and grid walking behavioral tests, as well as in the neurological disorder scores, in cerebral I/R rats. Moreover, BCAAO-induced inflammation was reduced by the administration of this drug, evidenced by reduced levels of cytokines IL-1β, TNF-α, and IL-6 and upregulation of IL-10. Additionally, membrane lipid peroxidation was reduced in the cerebral I/R rats receiving 100 mg/kg bupropion, and the level of SOD activity was improved in these animals. Finally, the administration of bupropion prevented the increase in NO_2_^-^ levels induced by BCAAO.

**Conclusions:**

In conclusion, bupropion has neuroprotective effects against cerebral I/R damage by reducing inflammation and oxidative stress in the brain.

## 1. Background

Brain stroke is a neurological disorder associated with damage to the central nervous system (CNS) and is classified into two types: Ischemic and hemorrhagic (1). Most cases of stroke result from ischemia, where the interruption of blood flow leads to the death of surrounding neurons due to impaired blood supply, causing disturbances in homeostasis and ion balance (2). This disruption, particularly during the reperfusion phase, leads to oxidative stress caused by free radicals, which in turn triggers the high secretion of inflammatory cytokines and activates cell death pathways, including apoptosis and necrosis (2). For instance, after cerebral ischemia/reperfusion (I/R) injury, the upregulation of IL-1 has been shown to result in severe damage (3), and IL-1β overexpression has been linked to neurodegeneration (4). Similarly, TNF-α (5) has been implicated, and its reduction through neutralizing antibodies resulted in a decrease in infarct size (6).

Thrombolytic drugs are the first line of treatment for ischemic stroke, associated with better clinical outcomes when administered promptly (7, 8). Studies have demonstrated that free radical scavengers can prevent brain I/R injuries (9). Bupropion, a dopamine reuptake inhibitor, is commonly prescribed for depression and smoking cessation (10). This drug increases the levels of dopamine and noradrenaline in the hippocampus and exerts therapeutic effects via nicotinic acetylcholine receptors (11). Bupropion’s anti-inflammatory effects have been documented in various studies, with its mechanism attributed to the inhibition of TNF-α secretion (12). Additionally, Camara-Lemarroy et al. found that bupropion inhibited the inflammatory cytokines TNF-α, IL-1, and IFN-γ, while enhancing the anti-inflammatory cytokine IL-10 in a rat model of intestinal ischemia-reperfusion (13). However, this drug had no effect on malondialdehyde (MDA) levels, indicating its ineffectiveness against oxidative stress induced by intestinal I/R injury (13). Furthermore, at a dose of 5 mg/kg (i.p.), bupropion prevented increases in MDA and nitrate levels and a decrease in reduced glutathione (GSH) levels in morphine-treated rats, suggesting its antioxidant activity (14). Bupropion has also been reported to improve enzymatic antioxidant defense (15). Therefore, it appears that this drug could be considered in other conditions, such as I/R injury, due to its anti-inflammatory and antioxidant activities.

## 2. Objectives

Given the significant roles of oxidative stress and inflammation in the pathophysiology and outcomes of cerebral I/R injury, and the reported anti-inflammatory and antioxidant effects of bupropion, the present study aimed to investigate the neuroprotective effects of bupropion against cerebral I/R damage in a rat model.

## 3. Methods

### 3.1. Animals and Grouping

Forty male Wistar rats (MW: 220 – 260 g) were obtained from Tehran University of Medical Sciences. The animals were transferred to the laboratory (Temp: 25 ± 2°C; RH: 55 - 65%; 12/12 h light and dark cycle) and housed in cages with free access to food and water. They were treated according to the ethical guidelines provided by the Animal Ethics Committee.

The rats were divided into four groups as follows:

(1) Control group: Rats received only normal saline (the solvent for bupropion) (n = 10).

(2) BCAAO group: Rats underwent bilateral common carotid artery occlusion (BCAAO) and received normal saline (n = 10).

(3) 60 mg/kg bupropion group: Rats underwent BCAAO surgery and received 60 mg/kg bupropion intraperitoneally (i.p.) 30 minutes before BCAAO (n = 10).

(4) 100 mg/kg bupropion group: Rats underwent BCAAO surgery and received 100 mg/kg bupropion i.p. 30 minutes before BCAAO (n = 10).

It is worth noting that the selection of bupropion doses was based on a pre-test experiment to determine the appropriate doses.

### 3.2. Induction of Cerebral Ischemia/Reperfusion Injury

First, the rats were anesthetized by an intraperitoneal injection of 45 mg/kg ketamine. A sagittal incision was then made along the neck midline, and the two common carotid arteries were separated from the vagal nerves. The arteries were occluded for 30 minutes using a clamp. After this, the reperfusion phase was initiated by removing the clamp, allowing for 60 minutes of reperfusion. The wound was then sutured, and the animals were transferred back to their cages. Animal behaviors such as grooming, drinking, and movement were monitored as indicators of recovery following surgery (16).

### 3.3. Behavioral Tests

We performed grid walking, modified neurological severity score (mNSS), and novel object recognition tests one day after the BCAAO surgery. These tests were conducted to evaluate the sensorimotor functions in the animals.

#### 3.3.1. Grid Walking Test

This test was conducted in an apparatus with a metal square grid (45 × 45 cm²; 2.5 cm above the floor) equipped with a camera on the floor, after disinfecting it with 70% ethanol. Each rat was allowed to walk freely on the grid, and the number of footfalls was recorded over a 60-second period (17).

#### 3.3.2. Novel Object Recognition

This test consisted 3 phases as following.

##### 3.3.2.1. Adaptive

This phase was conducted over two consecutive days (5 minutes each day), during which the animals were allowed to roam freely in a carbonate box (50 × 50 × 60 cm³) without any objects.

##### 3.3.2.2. Training

After the adaptation period, the rats were allowed to roam in boxes containing two identical objects (A and B) for 5 minutes.

##### 3.3.2.3. Testing

Sixty minutes after the training, the animals were allowed to roam for 5 minutes in boxes similar to the training phase, with the exception that the B object was replaced with a C object. The time spent finding the novel object was recorded, and the cognitive function of the animals was assessed according to the method described by Kang et al. (18).

#### 3.3.3. Modified Neurological Severity Score

This test was conducted based on the method of Chen et al. to evaluate the sensorimotor and balance functions of the rats. Scores ranging from 0 to 18 were assigned to each animal, where 0 indicated normal function and 18 represented the most severe deficit (19).

### 3.4. Biochemical Evaluations

The brain homogenates were prepared in a buffer containing KCl (10 mM), MgCl_2_ (1.5 mM), PMSF (0.1 mM), Hepes/KOH (20 mM), and a protease inhibitor according to the method described by Navarro and Boveris (20). The homogenates were then centrifuged at 12,000 g for 300 seconds.

We evaluated the levels of IL-1β, IL-6, IL-10, TNF-α, MDA, SOD, GSH, and catalase (CAT) in the brain homogenates of rats, and NO_2_^-^ in the serum.

IL-1β, IL-6, IL-10, and TNF-α levels were determined using ELISA kits (R&D Systems) following the manufacturer’s instructions.

MDA, as an indicator of lipid peroxidation, was measured using a commercial kit (Sigma, Germany) according to the manufacturer's instructions, with the optical density measured at 532 nm using a spectrophotometer.

The level of NO_2_^-^ was measured using the Griess reaction and read at 570 nm using an ELISA reader.

The activities of the antioxidant enzymes CAT and superoxide dismutase (SOD) were measured based on previously established methods (21, 22). The level of reduced glutathione in the rats' brain homogenates was measured using the method described by Jollow et al. (23).

### 3.5. Histopathological Evaluations

After extracting the brains, they were immediately placed in 4% paraformaldehyde (Sigma, USA) for 48 hours to fix. The brains were then sectioned into 5 µm thick slices using a microtome and stained using the hematoxylin-eosin method. The prepared slides were observed under an optical microscope (Nikon, Japan). Histopathological image interpretation was performed by an expert pathologist.

### 3.6. Data Analysis

The Kolmogorov-Smirnov test was used to evaluate the normal distribution of the data. Analysis was conducted using the ANOVA procedure followed by Tukey’s post hoc test, with a significance level of P < 0.05, in GraphPad Prism software. All data are presented as mean ± SEM.

## 4. Results

### 4.1. Behavioral Tests

**Figure 1.**
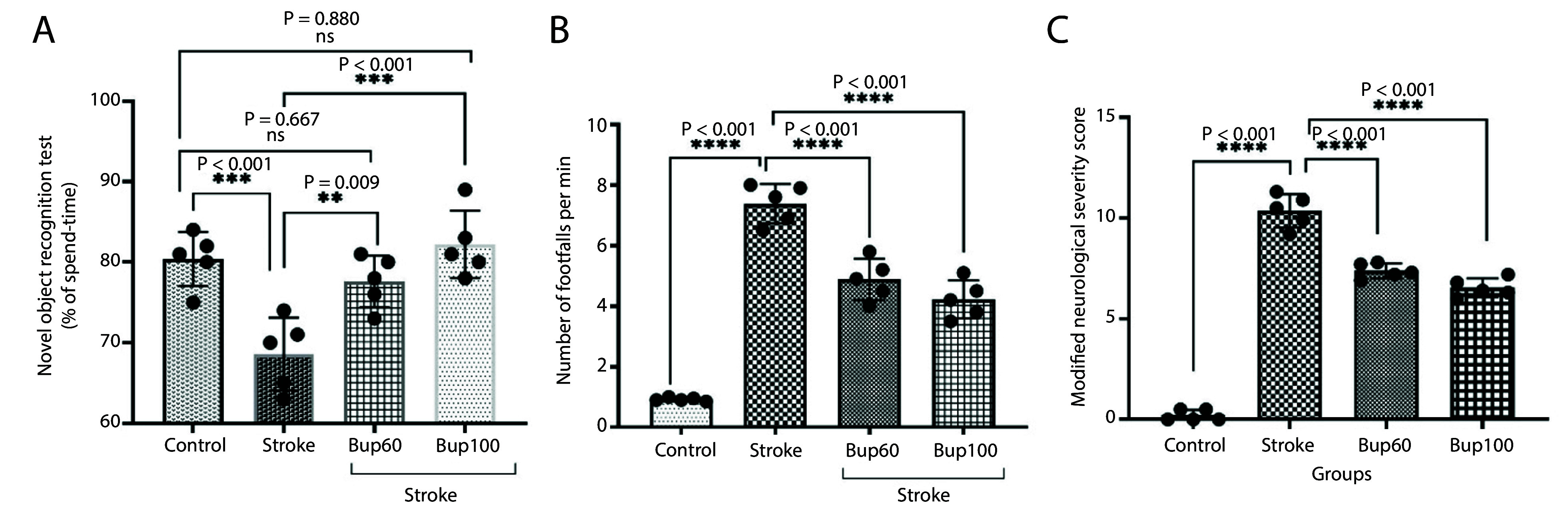
The effects of bupropion on the rats' sensorimotor function after BCAAO surgery in A, novel object recognition; B, grid walking; and C, modified neurological severity score (mNSS) tests. 60 and 100 mg/kg bupropion administrated ip 30 min before BCAAO surgery and the tests begun 24h after (n = 5).

The induction of cerebral I/R injury resulted in significant decreases in the Discrimination Index (approximately 13%) compared with healthy animals (Figure 1A). Additionally, the disease led to increases in the number of footfalls and mNSS scores, indicating reduced sensorimotor function in the animals (Figure 1B and C). Interestingly, rats pretreated with 60 and 100 mg/kg of the drug before the induction of the disease showed improvements in the Discrimination Index, as well as decreased numbers of footfalls and mNSS scores, indicating improved sensorimotor function.

### 4.2. Biochemical Evaluations

#### 4.2.1. Inflammatory Cytokines

**Figure 2.**
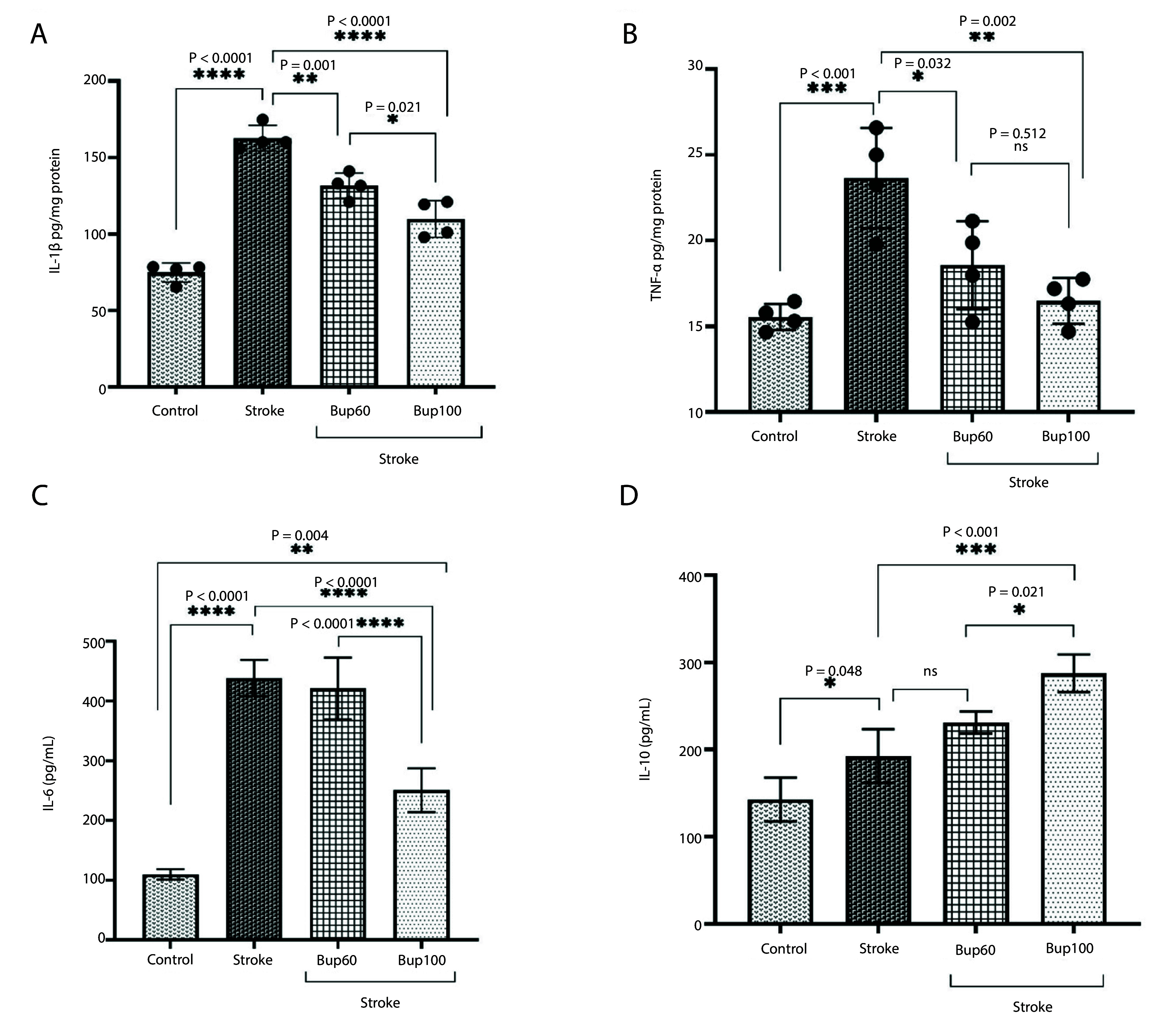
The effects of bupropion on the levels of A, IL-1β; B, TNF-α; C, IL-6; and D, IL-10 in rats' brains homogenates after BCAAO surgery. 60 and 100 mg/kg bupropion administrated ip 30 min before BCAAO surgery (n = 5).

IL-1β, TNF-α, and IL-6 were significantly overexpressed in the cerebral I/R injury rats compared to healthy animals. Similarly, IL-10 was also overexpressed, and the induction of disease resulted in increased levels of this cytokine. Pretreatment of cerebral I/R rats with 60 and 100 mg/kg bupropion significantly reduced the expression of both IL-1β and TNF-α (Figure 2A and B) compared with the positive control animals. However, IL-6 overexpression in cerebral I/R rats was only prevented in animals pretreated with 100 mg/kg of the drug (Figure 2C). Interestingly, pretreatment with both 60 and 100 mg/kg bupropion significantly increased the expression of IL-10 compared to untreated rats (Figure 2D). The highest expression of IL-10 was observed in the brains of I/R rats that received 100 mg/kg bupropion 30 minutes before disease induction. It is noteworthy that administration of 100 mg/kg bupropion resulted in a significant reduction in IL-1β (P = 0.021) and IL-6 (P < 0.0001), and significant increases in IL-10 (P = 0.021) levels compared to the cerebral I/R animals that received 60 mg/kg bupropion.

#### 4.2.2. Lipid Peroxidation and Antioxidant Enzyme Activities

**Figure 3.**
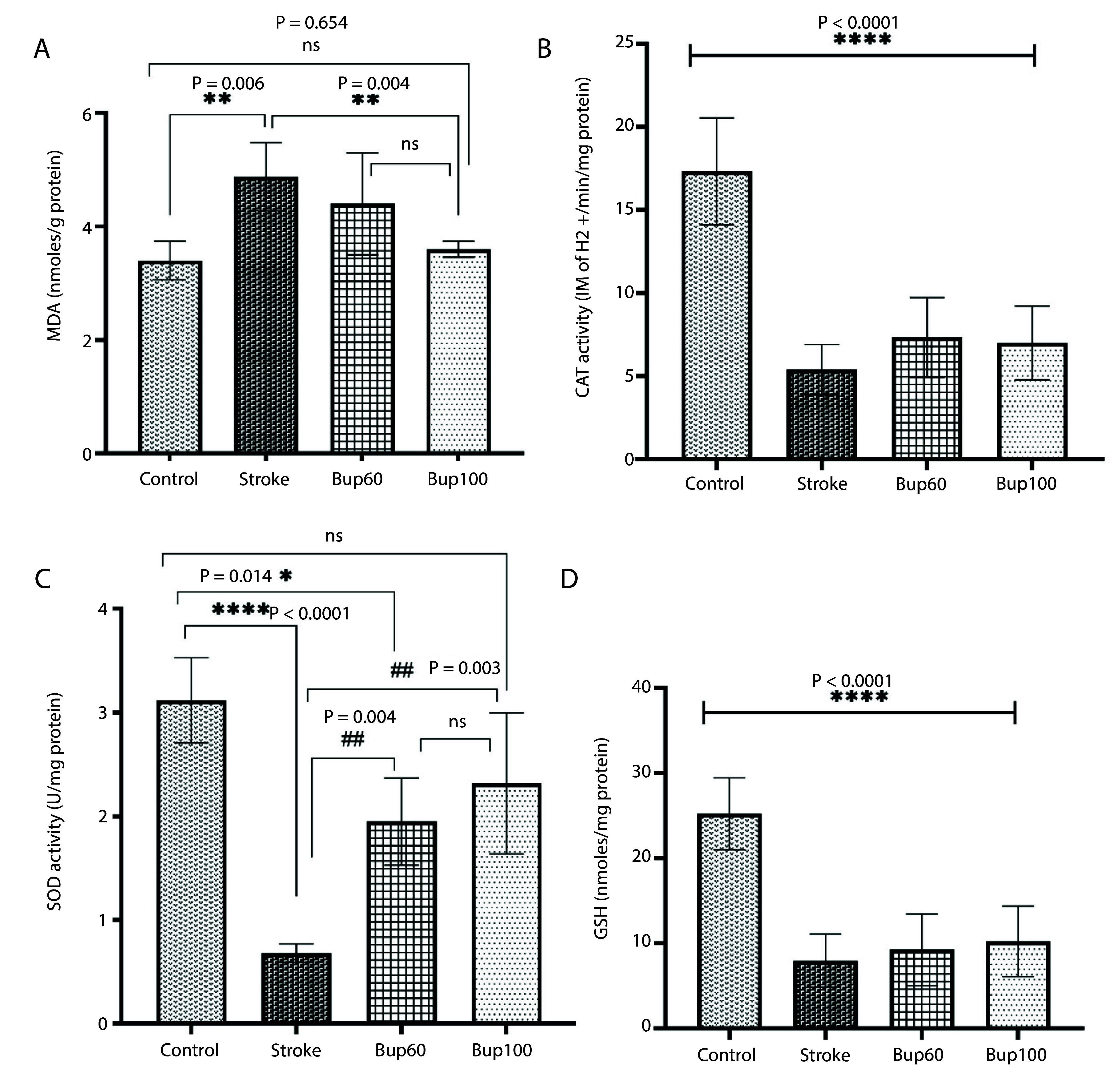
The effects of bupropion on the content of A, malondialdehyde (MDA); and the enzyme activities of B, catalase (CAT); C, superoxide dismutase (SOD) and reduced D, glutathione (GSG) in rats' brains homogenates after BCAAO surgery. 60 and 100 mg/kg bupropion administrated ip 30 min before BCAAO surgery (n = 5).

Cerebral I/R injury resulted in an increase in MDA content in the rats' brain homogenates, along with reductions in the activities of CAT and SOD enzymes and GSH content, indicating severe I/R-induced oxidative stress. Interestingly, pretreatment of rats with 100 mg/kg bupropion before cerebral I/R injury prevented the increase in MDA levels in the brain, showing no significant differences in MDA content between the control and cerebral I/R rats that received this drug (Figure 3A). Although there was no significant difference in CAT enzyme activity and GSH content in the brains of stroke group rats receiving bupropion compared to the positive control (stroke) rats (Figure 3B and D), a significant increase in SOD enzyme activity was observed in stroke rats receiving both 60 and 100 mg/kg of bupropion (Figure 3C). 

#### 4.2.3. NO_2_^-^

**Figure 4.**
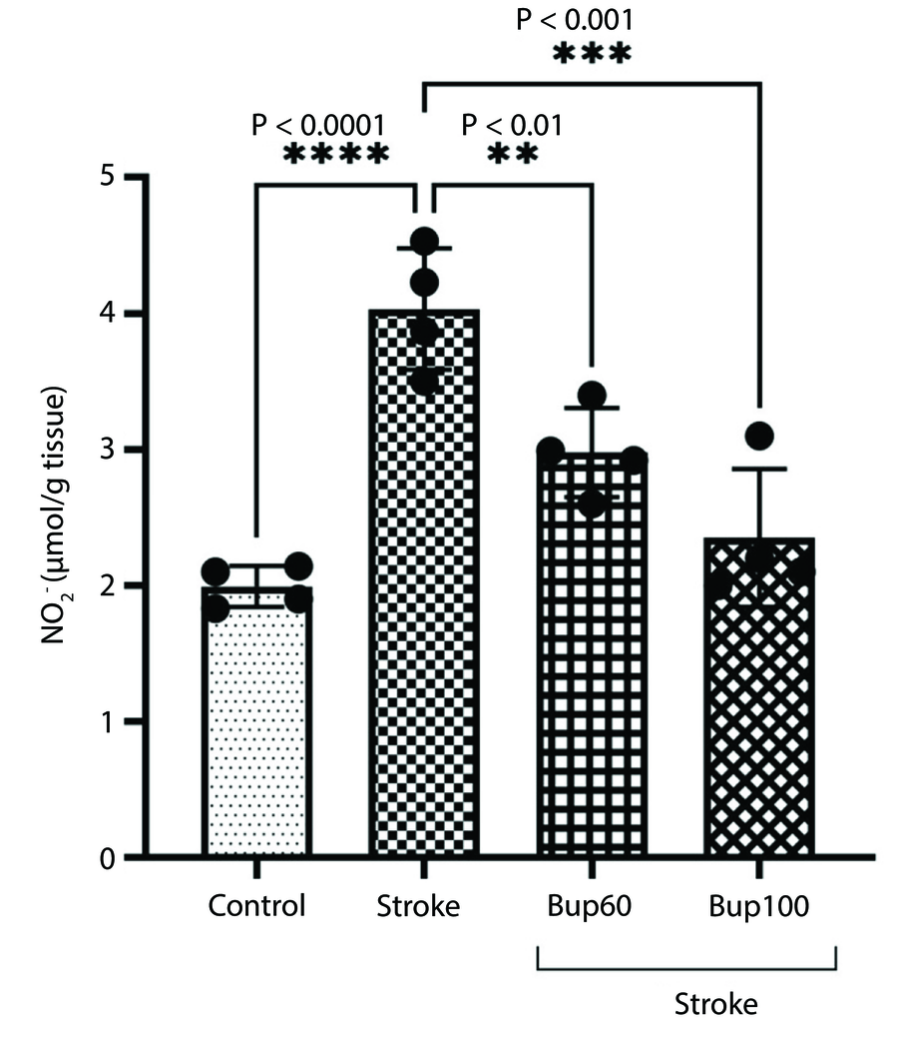
The effects of bupropion on the content of NO_2_^-^ in the rats' brains homogenates after BCAAO surgery. 60 and 100 mg/kg bupropion administrated ip 30 min before BCAAO surgery (n = 4).

We measured the level of NO_2_^-^ in the rats' brain homogenates, and the results showed significant increases in NO_2_^-^ levels in the cerebral I/R rats. However, bupropion prevented the increases in NO_2_^-^, such that no significant differences were observed in NO_2_^-^ levels between the ischemic rats administered 60 and 100 mg/kg of bupropion and the healthy rats (Figure 4). 

### 4.3. Histopathological Evaluations

**Figure 5.**
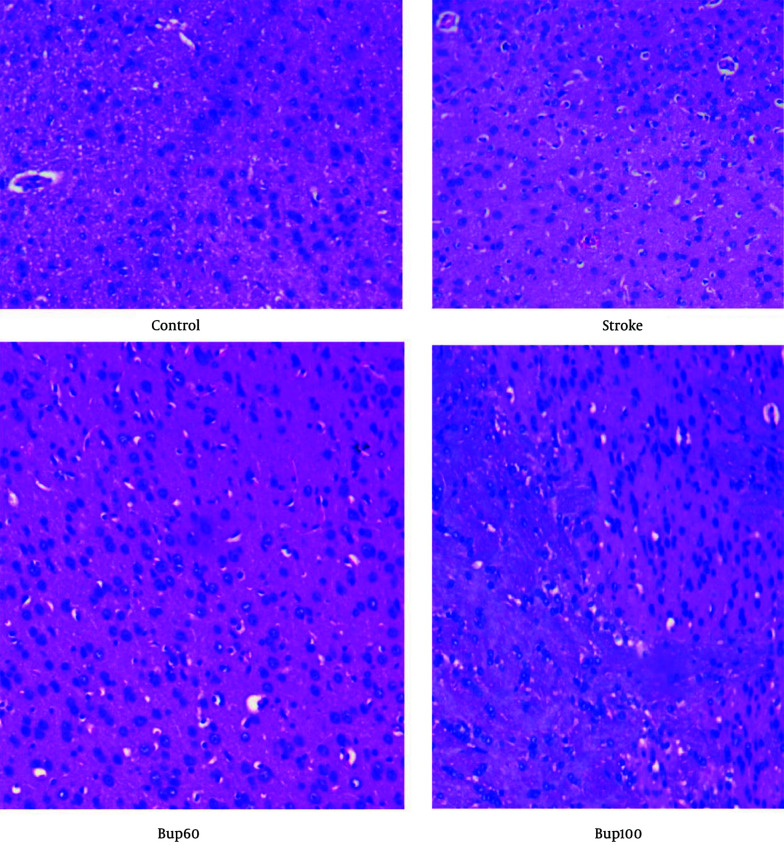
The histopathological evaluations of rats' brains in healthy and cerebral I/R injury groups received 60 and 100 mg/kg bupropion 30 min before the induction of disease (n = 3). The section brain slides were stained with H&E dyes and observed under optical microscopy (400 X).

Histopathological examination of the brain sections stained with H&E indicated that the neurons were normal without signs of neurodegeneration. However, in the stroke group, signs of neurodegeneration were evident, with neurons showing moderate pyknosis while oligodendrocytes were preserved. Additionally, moderate cerebral edema was observed in this group. In the brain of cerebral I/R rats treated with 60 mg/kg bupropion, moderate edema was observed in the white matter, but this was less severe compared to the positive control animals. Pyknotic neurons were still present, but there was less neuronal degeneration compared to the animals in the stroke group. Furthermore, histopathological examination of the brains of cerebral I/R animals receiving bupropion 30 minutes before induction of the disease showed less edema in the white matter compared to the stroke group. Active astrocytes, in combination with oligodendrocytes, were observed, with less neuronal degeneration and fewer pyknotic neurons compared to the stroke group (Figure 5). These results suggest the neuroprotective effects of bupropion against cerebral I/R injury.

## 5. Discussion

Cerebral I/R injury is one of the most debilitating diseases, associated with a significant decrease in quality of life and high mortality (24). Therefore, finding new treatment options that reduce or prevent the severity of this condition is of great importance. In this study, the effects of bupropion pretreatment were examined in a rat model of cerebral ischemia. This study, for the first time, demonstrated the neuroprotective effects of bupropion pretreatment against cerebral I/R damage. The drug improved the sensorimotor function of rats affected by cerebral I/R in all behavioral tests, and its mechanisms of action were attributed to the reduction of inflammation (downregulation of IL-1β, TNF-α, IL-6, and upregulation of IL-10) and a relative improvement in the cells' enzymatic antioxidant defense (increased SOD activity).

During cerebral I/R injury, overproduction of inflammatory cytokines occurs, leading to further damage to ischemic areas (25). Several studies have reported that IL-1β expression increases after ischemic injury, which is associated with significant damage to infarct areas (26-28). As a result, anti-IL-1β treatment has been recommended for patients, showing reduced damage (29). In the present study, an increase in the expression of IL-1β was observed after brain ischemia in rats, which is consistent with the findings of the aforementioned studies. Interestingly, IL-1β has the ability to regulate the expression of inflammatory cytokines such as TNF-α and IL-6. Therefore, the increase in IL-1β expression can be linked to the subsequent increase in TNF-α and IL-6 expression (30). Thus, the overexpression of TNF-α and IL-6 following ischemic injury in this study can be attributed to the overexpression of IL-1β. Notably, the administration of bupropion resulted in a decrease in the expression of IL-1β, TNF-α, and IL-6 in the brains of cerebral I/R rats, indicating the anti-inflammatory effects of this drug. Bupropion is known for its ability to inhibit TNF-α (12), and its ability to inhibit IL-1β has also been shown in the context of intestinal I/R damage (13). Interestingly, bupropion showed synergistic effects with celecoxib in reducing depressive symptoms in a mouse model by alleviating chronic inflammation (31). It was also recently demonstrated that the anti-inflammatory activity of bupropion is mediated through macrophages and the JAK2/STAT3 and TLR2/TLR4 pathways (32, 33). Bupropion administration has also been shown to reduce inflammatory biomarkers in severely depressed patients (34). In the present study, bupropion was shown to prevent the expression of inflammatory cytokines IL-1β, IL-6, and TNF-α in a cerebral I/R condition. These results confirm the anti-inflammatory effects of this drug.

IL-10 is a cytokine with anti-inflammatory properties that inhibits the expression of inflammatory cytokines, including IL-1β and TNF-α, as well as the NF-κB pathway (35). In the current study, the expression of IL-10 increased after the induction of cerebral I/R damage, likely indicating the body's response to the severe inflammation caused by the condition. Fouda et al. also demonstrated the upregulation of IL-10 after brain stroke in rats, although hypertension prevented this effect (36). It appears that increasing the expression of this cytokine could be a therapeutic approach for attenuating cerebral I/R-induced inflammation and subsequent injury. For example, the injection of 1 µg of IL-10 resulted in a reduction in infarct size in an ischemic stroke model in rats (37). In this study, bupropion significantly increased the expression of IL-10, which may explain the neuroprotective effects of bupropion in cerebral I/R damage observed in this research.

Cerebral I/R induction in this study increased MDA levels and decreased the activity of CAT and SOD antioxidant enzymes, as well as GSH content. These results indicate the induction of oxidative stress in the brains of cerebral I/R rats, which can lead to neurodegeneration due to the activation of apoptotic signaling pathways (38). The induction of oxidative stress in this condition is considered one of the key pathophysiological mechanisms underlying severe damage following cerebral I/R injury (38). Therefore, improving antioxidant status after cerebral I/R injury is regarded as one of the most important therapeutic approaches (25).

In the present study, administration of bupropion was associated with a significant decrease in MDA levels and a significant increase in SOD enzyme activity, indicating a relative reduction in oxidative stress in the brains of rats. The increase in SOD mRNA levels by bupropion has also been noted in another study (15), which aligns with the findings of the present study. However, the antioxidant activity of this drug is highly dose-dependent and may exhibit oxidant effects at high doses. For instance, exposure of SH-SY5Y cells to a high dose of this drug (100 μg/mL) resulted in Casp3 activation and endoplasmic reticulum stress, which was accompanied by activation of the apoptosis pathway and cell death (39). Furthermore, a recent study showed that high doses of this drug were associated with a decrease in mitochondrial complex II activity in isolated pig brain cells (40). Therefore, the neuroprotective effects of bupropion observed in this study can be attributed to the low dose and sub-toxic concentration of this drug.

The results of the present study indicate that bupropion could be considered as a therapeutic or adjunctive therapy for the treatment of cerebral I/R by reducing inflammation and oxidative stress. However, since the findings of this study were obtained in an animal model, caution should be exercised in generalizing the results to clinical settings. Clinical studies investigating the protective effects of this drug are strongly recommended for future research.

### 5.1. Conclusions

The pretreatment of bupropion in cerebral I/R rats resulted in less damage, as indicated by histopathological evaluations. The drug also improved the sensorimotor function of diseased rats, and the mechanism of action was attributed to its anti-inflammatory and relative antioxidant properties. However, further studies are needed in this regard.

## Data Availability

The dataset presented in the study is available on request from the corresponding author during submission or after publication.
